# Combinatory actions of CP29 phosphorylation by STN7 and stability regulate leaf age-dependent disassembly of photosynthetic complexes

**DOI:** 10.1038/s41598-020-67213-0

**Published:** 2020-06-24

**Authors:** Roshan Sharma Poudyal, Margarita V. Rodionova, Hyunmin Kim, Seongsin Lee, Eunjeong Do, Suleyman I. Allakhverdiev, Hong Gil Nam, Daehee Hwang, Yumi Kim

**Affiliations:** 1 Center for Plant Aging Research, Institute for Basic Science, Daegu, Republic of Korea; 2 K.A. Timiryazev Institute of Plant Physiology, Russian Academy of Sciences, Moscow, Russia; 3 Department of New Biology, DGIST, Daegu, Republic of Korea; 4 Present Address: Department of Agricultural Biotechnology, National Institute of Agricultural Science, Rural Development Administration, Jeonju, Republic of Korea; 5 Present Address: Department of biological sciences, Seoul National University, Seoul, Republic of Korea

**Keywords:** Photosynthesis, Plant sciences, Plant development, Leaf development

## Abstract

A predominant physiological change that occurs during leaf senescence is a decrease in photosynthetic efficiency. An optimal organization of photosynthesis complexes in plant leaves is critical for efficient photosynthesis. However, molecular mechanisms for regulating photosynthesis complexes during leaf senescence remain largely unknown. Here we tracked photosynthesis complexes alterations during leaf senescence in *Arabidopsis thaliana*. Grana stack is significantly thickened and photosynthesis complexes were disassembled in senescing leaves. Defects in STN7 and CP29 led to an altered chloroplast ultrastructure and a malformation of photosynthesis complex organization in stroma lamella. Both CP29 phosphorylation by STN7 and CP29 fragmentation are highly associated with the photosynthesis complex disassembly. In turn, CP29 functions as a molecular glue to facilitate protein complex formation leading phosphorylation cascade and to maintain photosynthetic efficiency during leaf senescence. These data suggest a novel molecular mechanism to modulate leaf senescence via CP29 phosphorylation and fragmentation, serving as an efficient strategy to control photosynthesis complexes.

## Introduction

Plant leaf senescence is the last and a highly organized stage of plant development^1,2^. Leaf senescence, as a post-mitotic senescence involving programed cell death, occurs after leaf maturation and involves multiple biological processes, including aging, hormonal responses, stress responses, altered catabolism, and transcriptional regulation.

This senescent phenotype is easily recognized as the leaves start to lose their green color and turn yellow as carotenoids are still remained. Leaf yellowing is thus the clearest visible indicator of the progress of leaf senescence and is caused by chlorophyll loss in the chloroplasts^3–5^. Chlorophyll loss is strongly associated with decreased photosynthetic efficiency; other catabolic processes follow in the chloroplasts during leaf senescence^6^. When the leaves are senescent, the chloroplast turns into an aged form, known as gerontoplast, which is enriched with plastoglobules - tubular and lipid-rich bodies - and in which the thylakoid membrane structure degenerates^7,8^. Gerontoplasts are the characteristic end products of leaf senescence, which involves decreased photosynthetic activity coupled with the remobilization of nutrients and their translocation to other developing or reproductive parts of the plant^9,10^.

The decrease in photosynthetic efficiency is also accompanied by a transition of leaves from sink to source organs. Photosynthetic efficiency is highest when the leaves are fully mature; subsequently, the efficiency declines gradually. One hypothesis suggests that decrease in photosynthetic efficiency in older leaves results from alterations in the photosynthetic apparatus during leaf senescence^11–13^. It is critical organizing photosynthetic protein complexes to modulate photosynthesis efficiency. External light is perceived by the light-harvesting complexes (LHC) of photosystem II (PSII) and photosystem I (PSI) and transferred to their reaction centers that relay electrons to proceeding photosynthesis. PSI, PSII, cytochrome b6f (Cyt b6f), and ATP synthase are the central components of electron transport process during photosynthesis. These basic protein complexes are selectively assembled in the thylakoid membrane in order to optimize photosynthetic efficiency^14^. PSII core accompanied by LHCII that is interlinked by Chlorophyll bound protein (CP) 24 (CP24), CP26 and CP29^15,16^. Irradiation of light triggers LHCII phosphorylation mostly through Ser/Thr protein kinase (STN) 7 (STN7) for migration of LHCII from PSII to PSI^17^. Whereas, another key Ser/Thr kinase, STN8 participated in repairing light-induced damages of the photosynthetic protein complexes phosphorylating majorly PSII core subunit proteins such as CP43, D2, D1 and PSBH^18,19^. Interestingly, the photosynthetic protein complexes exhibit differential rates of degradation during leaf senescence, with PSI decay occurring earlier than that of PSII^12^.

Several photosynthesis-related mutants of *Arabidopsis*, including mutants with defects in PSI protein components and several regulatory proteins involved in photosynthesis, were analyzed to determine whether they lead to an altered leaf senescence phenotype^20^. Defects in a number of PSI protein components led to a premature decrease in chlorophyll concentration in response to both natural and dark-induced senescence, suggesting that alterations in the photosynthetic apparatus can affect the leaf senescence process. Although it seems obvious that changes in chloroplast ultrastructure and decrease in photosynthetic rate are important to regulate leaf senescence, photosynthesis also affects leaf senescence. However, how these processes are linked and the key regulators controlling their interaction are unclear. In the present study, we investigated the molecular mechanism by which changes in chloroplast ultrastructure affect the photosynthetic protein complex during leaf senescence. To address chloroplast ultrastructure changes and their association with photosynthetic efficiency in response to leaf senescence, we investigated changes in chloroplast ultrastructure, which occur during leaf senescence, and identified that the key modulator is CP29 protein linking thylakoid structure with photosynthetic efficiency.

## Results

### Leaf age-dependent alteration of chloroplast ultrastructure

**Figure 1:**
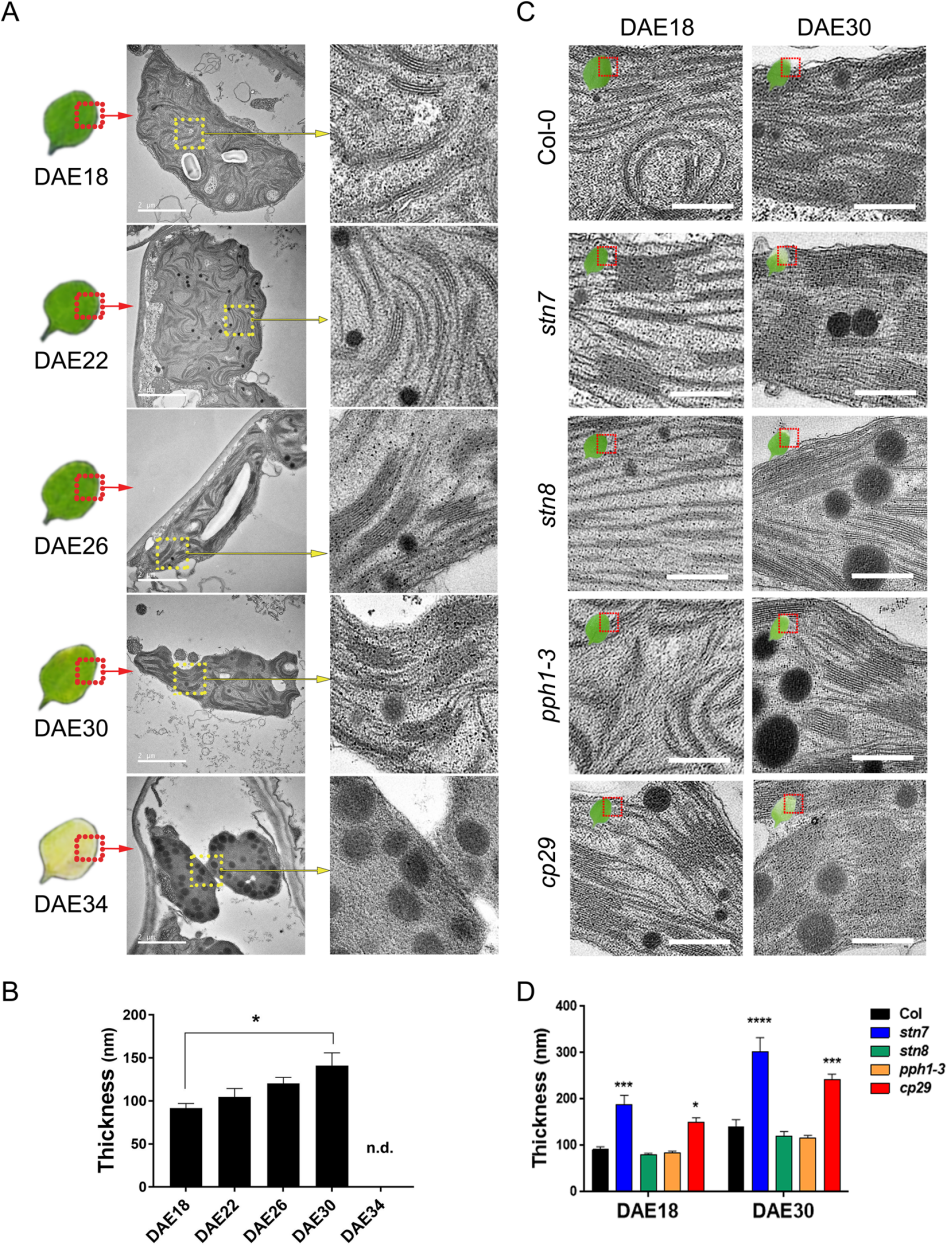
Grana stack thickness gradually increases with leaf age. (**A**) Chloroplast ultrastructure in aging *Arabidopsis* leaves. Representative *Arabidopsis* leaves were taken at each leaf age stage. Chloroplast images in red-dashed box and thylakoid membrane in yellow-dashed box were enlarged. White bars indicate 2 μm. **(B)** Quantitative measurement of the grana thickness from several hundreds of grana stacks. Leaves from DAE34 could not be determined owing to most of the chloroplast disruption. Data are mean ± SE from 3 biological replicates, and p-value was displayed after ordinary one-way ANOVA test. **(C)** Representative images of thylakoid membrane ultrastructure from WT, *stn7*, *stn8*, *pph1*-*3*, and *cp29* mutants at DAE18 and DAE30. Scale bars indicate 500 nm. **(D)** Measurement of grana thickness in WT, *stn7*, *stn8*, *pph1*-*3*, and *cp29* mutants at DAE18 and DAE30. Data are mean ± SE from 3 biological replicates, and p-value was displayed after two-way ANOVA test. Asterisks indicate *p < 0.05; **p < 0.01; ***p < 0.001; ****p < 0.0001.

Chloroplast ultrastructure was first analyzed in *Arabidopsis* leaves at five dates (Days after emergence (DAE) 18, 22, 26, 30, and 34) along leaf senescence using transmission electron microscopy to uncover the ultrastructure of chloroplast transition to gerontoplast. Structurally, mature leaves (DAE18) contained chloroplasts with multiple grana stacks, starch granules, and small plastoglobules. In contrast, senescent leaves (DAE34) showed enlarged plastoglobules with degenerated chloroplast outer and thylakoid membranes with typical features of gerontoplasts (Fig. 1A), indicating the transition of chloroplast to gerontoplast during senescence. Interestingly, the mean grana stack thickness in mature leaves (DAE18) was 91.57 ± 5.49 nm but progressively increased up to 141.15 ± 14.80 nm at DAE30 along the senescence (Fig. 1B).

Grana stack thickness is modulated under stress conditions by the phosphorylation of light harvesting complex (LHC) II in the thylakoid membrane as well as the amount of LHCI. Therefore, we examined the effects of phosphorylation of photosynthetic proteins in the thylakoid membrane on grana stack thickness using the mutants of the two major kinases (*stn7/stt7* and *stn8*) that phosphorylate LHCII and core components in PSII, respectively^17,18,21–23^ and a phosphatase (*pph1-3/tap38*) that dephosphorylates LHCII antenna^24–26^. LHCII contains three major proteins, Lhcb1, Lhcb2, and Lhcb3, and three minor components, including CP29, CP43, and CP47^27^. Previously, the depletion of the most abundant Lhcb1 and Lhcb2 was shown to have no defects in chloroplast^28^. Thus, we also included the null mutant of CP29 (*cp29*), a critical linker of electron flow between LHC and PSII core^29^, among the three CPs in PSII. Chloroplast ultrastructure in *stn7*, *stn8*, *pph1-3*, and *cp29* mutants was analyzed at DAE18 and DAE30 (Fig. 1C). Grana stack thickness in mature leaves (DAE18) of *stn7* (188.09 ± 20.08 nm) and *cp29* (153.97 ± 5.69 nm) was significantly greater than that in wild type (WT) mature leaves (P < 0.001 in *stn7* and P < 0.05 in *cp29*), similar to that in WT senescent leaves (DAE30). It further increased in senescent leaves of *stn7* (302.12 ± 30.80 nm) and *cp29* (241.94 ± 13.24 nm) to be almost two-fold thicker than that in WT senescent leaves (P < 0.0001 in *stn7* and P < 0.001 in *cp29*) (Fig. 1D). In contrast, grana stack thickness in *stn8* and *pph1-3* was comparable with those in WT at both DAE18 and DAE30. These data in *stn7* and *cp29* mutants suggest that these genes seem to be critical to modulate the chloroplast ultrastructure and have a tight association of the thickened grana stacks with leaf senescence phenotypes.

### Defects in STN7 and CP29 show an accelerated leaf senescence phenotype

**Figure 2:**
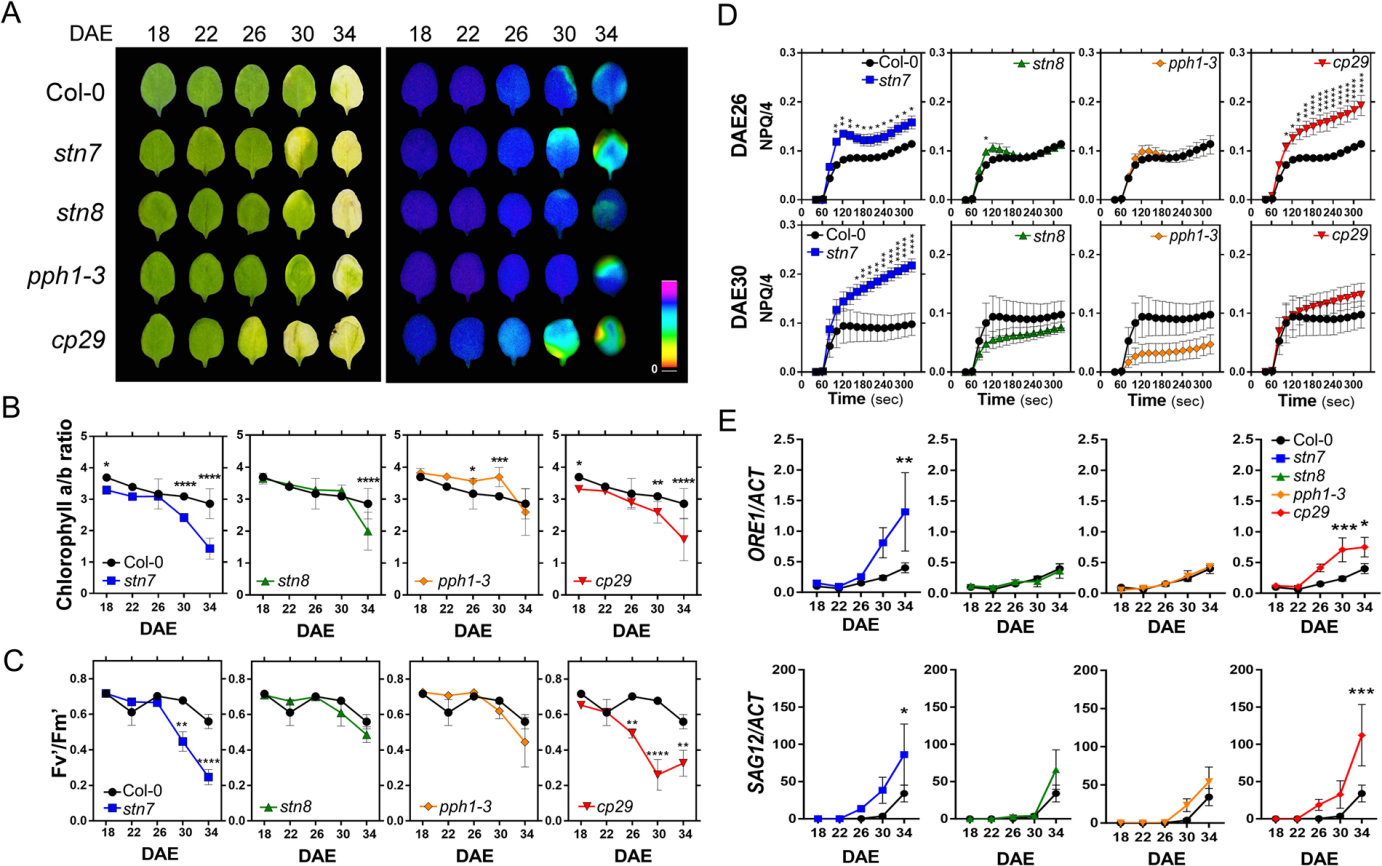
Defects in STN7 and CP29 showed accelerated leaf senescence phenotype. (**A**) Visual leaf senescence phenotypes and Fv’/Fm’ images of WT, *stn7*, *stn8*, *pph1-3*, and *cp29*. **(B)** Leaf age-dependent changes of chlorophyll a/b ratio. Data are mean ± SD from 8 leaves. **(C)** Measurement of the PSII maximum efficiency during leaf aging. **(D)** Nonphotochemical quenching during leaf aging. (C and D) Data are mean ± SE from 4 to 5 biological trials. **(E)** Age-associated gene expression during leaf senescence. Data are mean ± SE from 6 to7 biological trials. Asterisk indicates p-value after two-way ANOVA test (*p < 0.05; **p < 0.01; ***p < 0.001; ****p < 0.0001).

Thus, we next analyzed leaf senescence phenotypes in the mutants. The onset of leaf yellowing appeared to be clearly accelerated in *cp29* compared with that in WT, whereas *stn7, stn8* and *pph1-3* were indistinguishable from WT (Fig. 2A, left panel). Decreasing rate of total chlorophyll (Chl) contents in WT leaves during senescence showed no apparent differences with the other mutants we tested, except for *cp29* at DAE26, and carotenoid level stayed relatively stable during leaf senescence in WT and the other mutants (fig. S1). This suggests that early onset of leaf yellowing phenotypes in *cp29* was result of this Chl reduction. Further, we compared Chl a/b ratio, which indicates the size of LHC in the membrane^30,31^, along leaf senescence in the mutants. The decrease of Chl a/b ratio was accelerated from DAE26 and more significant at DAE34 in *stn7* and *cp29* compared with that in WT (Fig. 2B).

Moreover, we compared Chl fluorescence in PSII (*F*_*v*_’*/F*_*m*_*’*) among WT and the mutants after dark adaptation and nonphotochemical quenching (NPQ). Consistent with the observation from Chl a/b ratio, Chl fluorescence showed the same decreasing patterns in *stn7* and *cp29* (Fig. 2C). However, these patterns for Chl a/b ratio and Chl fluorescence were not apparent in *stn8* and *pph1-*3. Furthermore, we measured NPQ to examine the excess light emitted as heat^32^. NPQ increased at DAE22 and DAE30 in both *stn7* and *cp29*, respectively, compared with that in WT and then, became similar to that in WT at DAE34 (Fig. 2D and fig. S2). However, these increasing NPQ phenotypes were not observed in *stn8* and *pph1-*3. Leaf senescence marker genes, such as *ORESARA 1* (*ORE1*) and *SENESCENCE ASSOCIATED GENE 12* (*SAG12*), showed significantly earlier induction in *stn7* and *cp29* than in WT (P < 0.05 in *stn7* and P < 0.001 in *cp29*) (Fig. 2E). In contrast, such early induction patterns of these marker genes were not apparent in *stn8* and *pph1-3*. Taken together, all these data suggest that *stn7* and *cp29* regulate photosynthetic efficiency associated with leaf senescence.

### Photosynthetic protein complexes disassembled upon leaf senescence

Consistent decreases in Chl a/b ratios and Chl fluorescence, and leaf senescence phenotypes in *stn7* and *cp29* (Fig. 2) suggest that the proper organization of photosynthetic protein complexes might be important for effective photosynthesis^26,33,34^. Thus, we analyzed the compositions of Chl-protein complexes in senescing leaves of WT, *stn7*, *stn8*, *pph1-3*, and *cp29* after dissolving thylakoid membrane using two previously reported detergents, n-dodecyl-3-D-maltoside (DM) and digitonin, for preferentially dissolving protein complexes in the grana and stroma lamella, respectively^14^. In the grana stack dissolved by DM, photosynthetic mega-complexes, PSI/PSII dimer, ATP synthase, PSII monomer/Cytb6f, LHCII assembly, LHCII trimer, and LHCII monomer were detected (fig. S3). These complexes were found to be degraded during leaf senescence, consistent with previous findings^12^; however, the composition of the complexes was not different in all mutants compared with that in WT (fig. S3).

**Figure 3:**
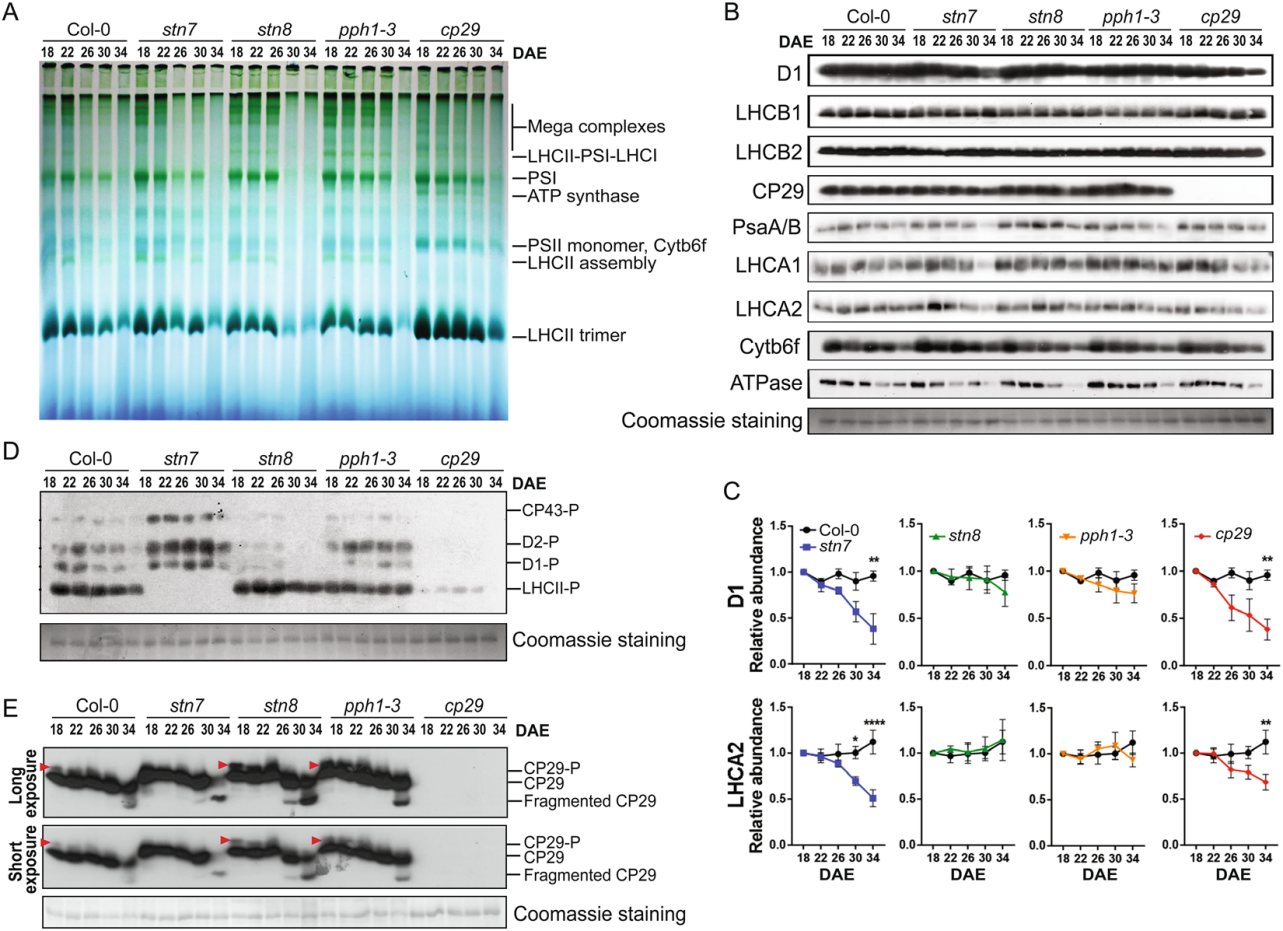
STN7 dependent CP29 phosphorylation and CP29 degradation are highly associated with leaf-age disassembly of photosynthetic protein complex in stroma lamella. (**A**) Age-dependent degradation of photosynthetic protein complexes in the stroma lamella. This blue native gel is a full length gel image. **(B)** Photosynthesis-related protein abundance along with leaf age. **(C)** Quantification of their abundance from 3 biological trials. Data are mean ± SE, and p-value was displayed after two-way ANOVA test. Asterisks indicate **p < 0.01 and ****p < 0.0001. (**D**) Leaf age-dependent decrease of phosphorylation of Ser/Thr residues in key photosynthetic proteins. Blot images of (D) and (**E**) were from a full-gel image each. (**E**) Changes in CP29 protein phosphorylation and fragmentation in senescing leaves. Phosphorylated CP29 (CP29-P) bands are indicated with red-arrows.

On the other hand, protein complexes in the stroma lamella enriched by dissolving with digitonin, photosynthetic mega-complexes, LHCII-PSI-LHCI, PSI, ATP synthase, PSII monomer/Cytb6f, LHCII assembly, and LHCII trimer were detected in WT leaves, and their amounts decreased during leaf senescence as in the grana stack (Fig. 3A). Remarkably, in the stroma lamella, we observed distinct alterations of these complexes in *stn7* and *cp29*. LHCII-PSI-LHCI complex was missing in *stn7*, even under growth in light conditions, which could cause defects in the phosphorylation of LHCII and movement of LHCII from the grana stack to stroma lamella^17,21^. Moreover, mega-complex formation and LHCII assembly were decreased in *cp29*, whereas ATP synthase, PSII monomer/Cytb6f, and LHCII trimer were increased (Fig. 3A and fig. S3). These data suggest that the defects in STN7 and CP29 lead to marked changes in the composition of photosynthetic complexes in the stroma lamella but not in the grana stacks.

### CP29 phosphorylation and fragmentation are highly associated with leaf senescence

We then examined whether the distinct alterations of photosynthetic protein complexes in *stn7* and *cp29* may be due to the altered levels of proteins associated with the complexes. Accordingly, we measured the amounts of several key proteins of the photosynthesis machinery, such as D1, LHCB1, LHCB2, and CP29 in PSII; PsaA/B, LHCA1, and LHCA2 in PSI; Cytb6f and ATPase in the thylakoid membranes of WT and the mutants. In WT leaves, among these proteins, only ATPase and Cytb6f levels decreased during leaf senescence (Fig. 3B and fig. S4). Interestingly, D1 in the PSII reaction center and LHCA1/LHCA2 in PSI selectively and dramatically decreased in both *stn7* and *cp29* leaves during leaf senescence compared to that in WT leaves but not in *stn8* and *pph1-3* leaves (Fig. 3C). Thus, the degradation of associated proteins in the photosynthetic complexes are not a major cause of the Chl-protein complexes decay during leaf senescence, and it is not clear how the selective decreases of D1 and LHCA1/LHCA2 in *stn7* and *cp29* during the senescence are linked to the photosynthetic protein complexes showing the distinct alterations in these mutants.

The phosphorylation of proteins in the photosynthesis machinery by two Ser/Thr kinases, STN7 and STN8, is prominent^18^. Thus, we next examined whether the distinct alterations of protein complexes in *stn7* and *cp29* may be associated with the altered phosphorylation of photosynthetic proteins. Accordingly, we measured the phosphorylation levels of the four most abundant phosphorylated proteins (CP43, D1, D2, and LHCII) in the photosynthetic machinery using the thylakoid membranes from WT and the mutants. The phosphorylation of all the four proteins decreased in WT leaves along the senescence (Fig. 3D). In turn, decreased photosynthesis protein complexes in the thylakoid membrane correlated with reduced phosphorylation levels rather than with the abundance of key proteins in the complexes. Surprisingly, although CP29 is not a kinase, all these proteins showed markedly reduced phosphorylation in *cp29*.

Further, we examined the phosphorylation changes of CP29 using the thylakoid membranes from WT and the mutants along the senescence. Phosphorylated-CP29 was detected right above the main CP29 protein band and decreased along leaf senescence, and additionally fragmented CP29 proteins were increased in senescing leaves. Interestingly, unlike in WT and *stn8* and *pph1-3*, no phosphorylated bands of CP29 were found in *stn7* along the senescence (Fig. 3E and fig. S6). These data suggest that the direct phosphorylation of CP29 by STN7 and both the lack of CP29 phosphorylation and absence of CP29 are highly associated with the altered organization of photosynthetic protein complexes in *stn7* and *cp29*.

### Contribution of CP29 isoforms to photosynthetic protein complexes

**Figure 4:**
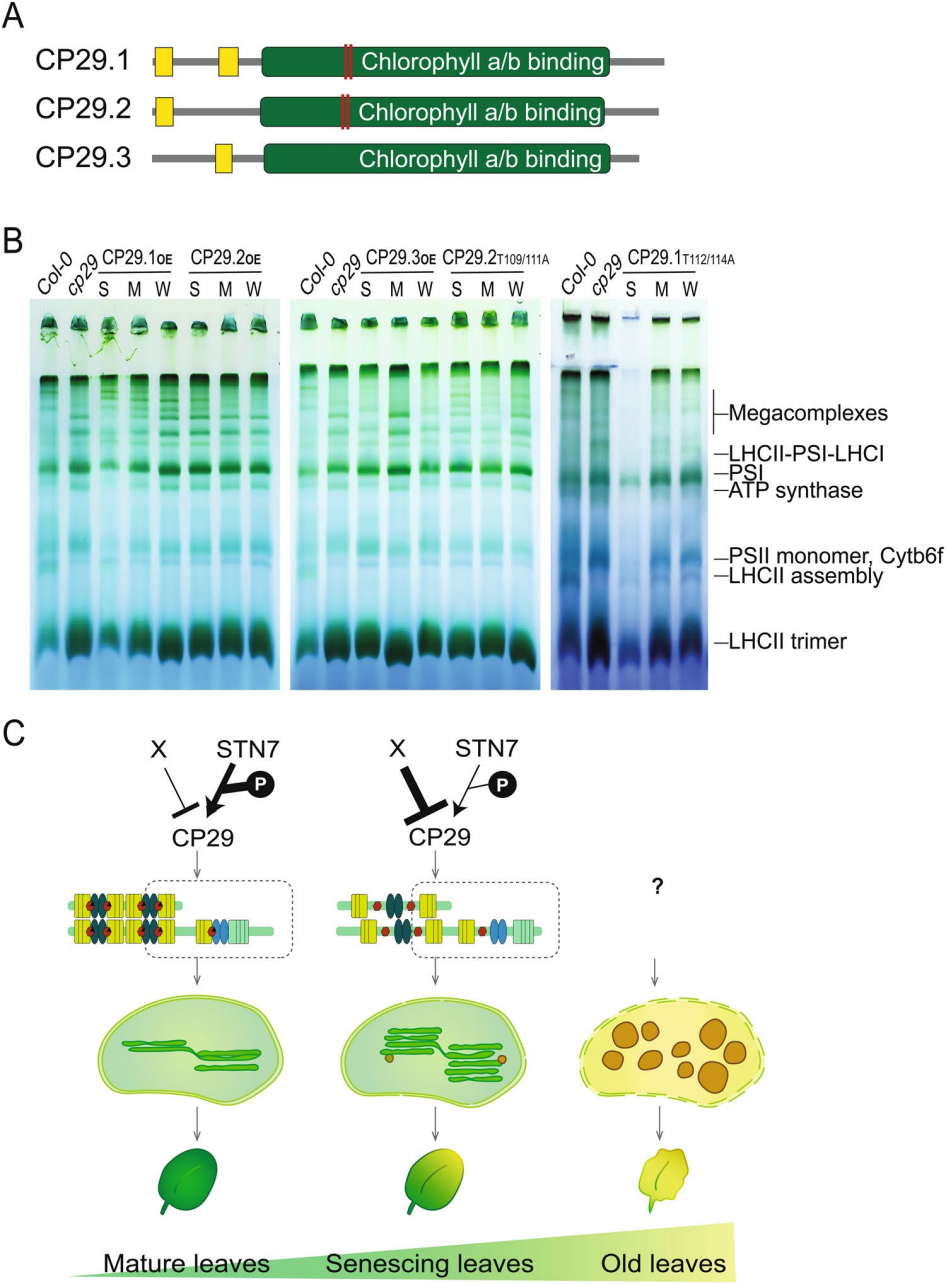
CP29.1 and CP29.2 are sufficient to reconstitute photosynthetic protein complex. (**A**) Protein structure of three CP29 isoforms. Yellow boxes represent a low-complexity region and red lines indicate known phosphorylation sites. **(B)** CP29 dosage-dependent complementation of photosynthetic protein complexes. Thylakoid membrane which expresses strong (S), medium (M), and weak (W) protein levels of CP29 isoforms were loaded. These samples were run in three different gels with WT and *cp29* controls. **C)** Working model for the molecular mechanism of STN7 and CP29 regulating photosynthetic protein complex organization during leaf senescence.

In *Arabidopsis, Lhcb4.1*, *Lhcb4.2*, and *Lhcb4.3* encode three CP29 isoforms, CP29.1, CP29.2, and CP29.3 proteins, respectively, with amino acid sequence identity from 67% to 89% (fig. S7). Compared with *Lhcb4.3, Lhcb4.1* and *Lhcb4.2* are more highly expressed^35,36^. The three isoforms contain chlorophyll A/B binding domains in C-terminus and low-complexity regions in N-terminus, which are diversified from each other (Fig. 4A). CP29 is phosphorylated by STN7 in the chlorophyll A/B binding domain at Thr112 and Thr114 in CP29.1; Thr37, Thr109, and Thr11 in CP29.2; and Ser34 in CP29.3^37–39^. Further, based on sequence comparison, Thr112 and Thr114 in CP29.1 and Thr109 and Thr111 in CP29.2 were revealed as conserved amino acids, which were absent in CP29.3 on the chlorophyll A/B binding domain (Fig. 4A and fig. S7).

To examine the effects of CP29.x’s abundance on the photosynthetic protein complex organization, three transgenic lines overexpressing *Lhcb4.1* (CP29.1_OE_), *Lhcb4.2* (CP29.2_OE_), and *Lhcb4.3* (CP29.3_OE_) genes were generated, respectively. Further, transgenics for testing effects of CP29.1 and CP29.2 phosphorylation in two conserved phosphorylation sites, two conserved threonine residues were substituted into alanine and overexpressed in *cp29*, named as CP29.1_T112/114A_ or CP29.2_T109/111A_, respectively. For each transgenic line, we tested 24 transformants and then, selected three lines with strong, mild, and weak expression of the corresponding CP29 isoform to examine the dosage-dependent complementation (fig. S8). CP29.1_OE_ and CP29.2_OE_ showed reconstitution of the mega-complexes in dosage-dependent manners, but CP29.1_T112/114A_ and CP29.2_T109/111A_ showed much weaker reconstitution (Fig. 4B), suggesting positive regulation of the mega-complex formation by phosphorylation of CP29.1 and CP29.2 at the conserved phosphorylation sites. In contrast, CP29.3 barely reconstituted the mega-complexes. Collectively, these data suggest that both phosphorylated CP29 and mainly its abundance of CP29.1 and CP29.2 function as molecular glues to facilitate photosynthetic protein complex formation, thereby maintaining photosynthetic efficiency in mature leaves.

## Discussion

The decreasing efficiency of photosynthesis is a widely known feature in plant leaf senescence, but its molecular mechanism is largely unknown. As we firstly observed and quantified, grana stacks were gradually thickened before the chloroplast turns into gerontoplasts. This changing chloroplast ultrastructure during leaf senescence in WT and mutants is highly correlated with their senescence phenotypes. Further, the dissociation of photosynthetic protein complexes without the reduction of key photosynthesis protein components in both the grana and stroma lamella enriched membrane fractions was also tightly associated. This implies that photosynthesis complexes disassembly can trigger leaf senescence because it was observed prior to visual and molecular senescence phenotypes. Especially, the organization of photosynthetic protein complexes in the stroma lamella was altered in *stn7* and *cp29* mutants which may be a cause of accelerated senescence phenotypes in those mutants. Comparing chloroplast ultrastructure with photosynthetic protein complex formation in *stn7* and *cp29* mutants (Fig. 1, Fig. 3A and fig. S3), there can be found that the grana stacks were greatly thickened, but the altered photosynthetic protein composition has been observed only in stroma lamella enriched thylakoid membrane fraction in both mutants. A possible explanation for this would be that phosphorylation of LHCII seems to be highly responsible for thylakoid membrane stacking, since it showed dramatic decrease of LHCII phosphorylation both in *stn7* and *cp29*. Further, reduced LHCII phosphorylation might perturb their movement to stroma lamella to form further complexes of photosynthesis such as LHCII-PSI in stroma lamella in *stn7* and *cp29* mutants.

STN7 is a major kinase for CP29 phosphorylation under growth condition which is a distinguishable mechanism from high-light dependent CP29 phosphorylation by STN7 for PSII supercomplex disassembly^40^. We found that the combinatory action of CP29 abundance control together with its phosphorylation by STN7 is critical for the organization of photosynthesis protein complexes by the complementation of each CP29 isoform and with their known phosphorylation site substitutions. This process seems to be temporally regulated because *stn7* and *cp29* mutants have no significant alterations of photochemical efficiency in mature leaves. Further, D1 and LHCAs were significantly decreased in *stn7* and *cp29* mutants, and this decrease may be in line with age-dependent STN7-CP29 phosphorylation pathway, which still needs to be studied.

Isoforms of CP29 - CP29.1 and CP29.2 - were sufficient to reconstitute the photosynthetic protein complexes, and it seems that there are additional leaf senescence-dependent CP29 phosphorylation sites by STN7 other than the two conserved phosphorylation sites that we tested. CP29 fragmentation was found to occur as leaves age, and this degradation looked clearly independent of STN7, STN8, or PPH1, which indicates CP29 degradation goes through novel pathways with leaf senescence dependent manner (Fig. 4C).

Notably, CP29, a Chl-bound light harvesting protein, is not a kinase, yet the phosphorylation of major reaction center proteins and LHCs were significantly diminished in *cp29* mutant. This is an intriguing phenomenon since the result indicates that a structural role of CP29 linking LHCII with PSII core unit, a proper organization of protein components for photosynthesis is critical for leading phosphorylation cascades in the thylakoid membrane. Possibly, leaf senescence might be initiated by loss of phosphorylation mainly in LHCs accompanied with an altered thylakoid membrane ultrastructure. In conclusion, the temporal regulation of photosynthesis protein complex organization by STN7 and CP29 triggers leaf senescence that leads to the transition of chloroplasts to gerontoplasts. This study provides an initial and novel molecular mechanism to elucidate leaf senescence-dependent decrease in photosynthetic efficiency.

## Materials and Methods

### Plant materials and growth condition

*Arabidopsis thaliana* ecotype Columbia (Col-0) was used as wild-type (WT). Similarly, single transgenic mutants used in this study were described previously as follows: *stn7*^41^, *stn8*^18^, *pph1-3*^42^, and *cp29*^27^. Plants were grown in soil at 22 °C ± 1 °C with 100 µmol photons/m^2^s, 16 h /8 h of day night photoperiod and 70% relative humidity. The third and fourth leaves were used for the entire experiment on days after emergence (DAE) of leaf primordia from DAE 18, 22, 26, 30, and 34.

### Observation of chloroplast ultrastructure

The 3^rd^ and 4^th^ leaves of *Arabidopsis* plants were collected accordingly and prepared for transmission electron microscopy (TEM)^43^. Three leaves were used for each trial and right-upper quadrant of leaves were targeted for image collections. At least 12 chloroplasts and 300 grana stacks from each leaf were used for quantifying the thickness of grana size. Grana height were measured by using Image J software.

### Quantification of chlorophyll and carotenoid contents

The fresh weight of leaves was recorded before grinding tissues in liquid nitrogen. The pigments were extracted using 80% acetone. Leaf debris was removed by centrifugation at 13,000 rpm (Centrifuge 5424 R, Eppendorf, Germany) for 10 min at room temperature. The supernatant was used, and absorbance was recorded at 470 nm, 646.6 nm, 663.6 nm and 750 nm. Finally, chlorophyll *a*, chlorophyll *b*, total chlorophyll (*a* + *b*), chlorophyll a/b ratio and carotenoid was calculated according to previous research^44,45^.

### Analysis of photosynthetic parameters and performance

Fresh leaves were harvested and placed in blocks wrapped in water-moistened filter paper for 15 min of dark incubation. Photosynthetic performance was analyzed using IMAGING-PAM M-Series (Heinz Walz GmbH, Effeltrich, Germany). The effective quantum yield of photosystem II (PSII) and non-photochemical quenching (NPQ) were then recorded according to the manual.

### Semiquantitative measurement of senescence associated genes

Expression of senescence associated genes (SAGs) was analyzed using qRT-PCR. Total RNA was extracted from leaf tissues using TRIzol (Invitrogen, USA), and cDNA was synthesized using ImProm-II Reverse Transcription System (Promega, USA) as the template for subsequent PCR amplification with SYBR (BioRad, USA). Expression of *ORE1* and *SAG12* were analyzed as described in Kim *et al*. (2018).

### Isolation of thylakoid membranes and proteins

Thylakoid membranes were isolated from frozen leaves according to Järvi *et al*.^14^. Total chlorophyll content in an aliquot of thylakoid membrane was determined using the method provided by Porra *et al*.^44^. Total protein content in the thylakoid membrane was extracted using protein extraction buffer (0.1-M Tris-HCl pH 7.6, 4% SDS and 1x protease cocktail, Roche Life Science, Germany), and total protein was quantified according to BCA method (Thermo Scientific, USA).

### Blue native polyacrylamide gel electrophoresis (BN-PAGE)

Thylakoid membranes were isolated from frozen leaves according to Järvi *et al*.^14^. Two different methods of BN-PAGE were performed to isolate different thylakoid membrane protein complexes. First, to separate photosynthetic protein complexes from the grana stack, an aliquot of thylakoid membrane containing 20 µg of chlorophyll was solubilized in 0.5% n-dodecyl β-D-maltoside. Similarly, to separate photosynthetic protein complexes from the grana margin and stroma lamella of thylakoid membrane, thylakoid membrane containing 20 µg of chlorophyll was solubilized in 1% digitonin and BN-PAGE was performed using the procedure described by Järvi *et al*.^14^.

### Protein gel blot assay

Protein gel blot assay was performed according to Towbin *et al*.^46^. Accordingly, 5 µg of thylakoid membrane protein was denaturized by boiling with SDS sample buffer for 5 min and separated by SDS-PAGE using 15% (w/v) acrylamide gel. PsbA (D1 protein), Lhcb1, Lhcb2, Lhcb4 (CP29 protein), Lhca1, Lhca2, Cyt*b6f*, and ATPase were detected using corresponding antibodies provided by Agrisera, Sweden. PasA/B protein was detected by using an antibody that was used in Nath *et al*.^12^. Phosphorylation of PSII proteins and LHCII proteins were detected using phosphothreonine antibody (Cell Signaling, USA). For separation of phosphorylated and fragmented CP29, Phospho-tag (NARD Institute, ltd. Japan) was added into 15% acrylamide gel according to the manufacturer’s instruction.

### Generation of CP29 transgenic plants

Three isoforms of CP29 gene (*lhcb4.1, lhcb4.2*, and *lhcb4.3*) were amplified from cDNA of *A. thaliana* Col-0 (DAE 18) using following primers:

**Table:** 

LHCB4.1	F	ACT GTC GAC ATG GCC GCA ACA TCC GCC GCT GCT
	R	GAA GCG GCC GCT TAA GAT GAG GAG AAG GTA TCG ATG
LHCB4.2	F	ACT GTC GAC ATG GCC GCC ACT TCA ACC GCC GCT
	R	GAA GCG GCC GCT CAG GAG GAA GAG AAG GTA TCG ATA
LHCB4.3	F	ACT GTC GAC ATG GCT ACC ACC ACT GCA GCA GCA
	R	GAA GCG GCC GCC TAA TTG TTA AAG GTG GCA AGG AAG

The amplicon was inserted to pCR-CCD vector, containing the *ccdB* gene, and transferred into *E. coli* TOP10 competent cells. Cells were selected on LB agar plates containing spectinomycin. The plasmid insert was confirmed by sequencing.

To substitute phosphorylating threonine residues by nonphosphorylating alanine residues, site-directed mutagenesis was performed using Q5 site-directed mutagenesis kit (New England Biolabs, USA) with primers listed below:

**Table:** 

CP29.1_T112/T114	F	CGGAgCCCGTgCGGAAGCTGC
CP29.1	R	ATCACGTCTCCGGCCAAGTTC
CP29.2_T109/111	F	CGGGgCTCGTgCCGAGGCGGT
CP29.2	R	ATCACTTCTCCGTATAAGTTCTTGGCTAAGTTCTG

The amplification, ligation, and transformation of *E. coli* competent cells were performed according to the manufacturer’s protocol. Cells were selected on LB agar plates containing antibiotic spectinomycin. The substitutions of amino acids in positions C29.1 T112/114 and CP29.2 T109/111 were confirmed by sequencing.

Next, Gateway LR reaction was performed with pCR-CCD::LHCB4 and pCR-CCD::LHCB4-mutants and pCSV-1300 destination vector using Gateway LR Clonase II Enzyme Mix (Thermo Fisher Scientific, USA) according to the manufacturer’s protocol. Reactions were transformed into *E. coli* TOP10 cells and selected on LB agar plates with kanamycin. The LR reaction was confirmed by sequencing with CsV-promoter primer 5′-GAAGTACTGAGGATACAAC-3′.

Purified pCSV-1300::LHCB4 and pCSV-1300::LHCB4-mutant plasmids were transferred into agrobacteria AGL-1 by electroporation. Agrobacteria were selected on LB agar plates containing rifampicin and kanamycin, cultured in YEP medium, and cells were then harvested by centrifugation and resuspended in 1x B5 floral dipping solution. The floral dip transformation method was applied to *cp29* knockout mutants. The transgenic seeds (T1) were planted on 1/2 B5 agar plate containing hygromycin and cefotaxime. Further, the transgenic plants were transferred to soil for further analysis.

## Supplementary information

Supplementary Information.

## Data Availability

All data is available in the main text or the supplementary materials.
