# Supplementary material for: Combinatory actions of CP29 phosphorylation by STN7 and stability regulate leaf age-dependent disassembly of photosynthetic complexes
Source: Sci Rep. 2020 Jun 24;10:10267. doi: 10.1038/s41598-020-67213-0 (PMC7314821; doi:10.1038/s41598-020-67213-0)
Supplement: Supplementary file 1 — Supplementary Information. [file 41598_2020_67213_MOESM1_ESM.docx]

Supplementary materials

Combinatory actions of CP29 phosphorylation by STN7 and stability regulate leaf age-dependent disassembly of photosynthetic complexes

Roshan Sharma Poudyal^1,†^, Margarita V. Rodionova^2†^, Hyunmin Kim^1^, Seongsin Lee^1^, Eunjeong Do^1^, Suleyman I. Allakhverdiev^2,3,4^ , Hong Gil Nam^1,4^*, Daehee Hwang^1, 5,^ * and Yumi Kim^1, †,^*

†: These authors are equally contributed to this work.

*Correspondence to: nam@dgist.ac.kr, daehee@snu.ac.kr, yumikim@ibs.re.kr

**This PDF file includes:**

Supplementary Figures 1 to 12

**Supplementary Figure 1** Changes in total chlorophyll and carotenoid contents. Total chlorophyll (A) and carotenoid (B) contents were measured along with leaf aging. Data are mean ± SD from 8 leaves and asterisk indicates p-value after two-way ANOVA test (*, p < 0.05; **, p < 0.01; ****, p < 0.0001).


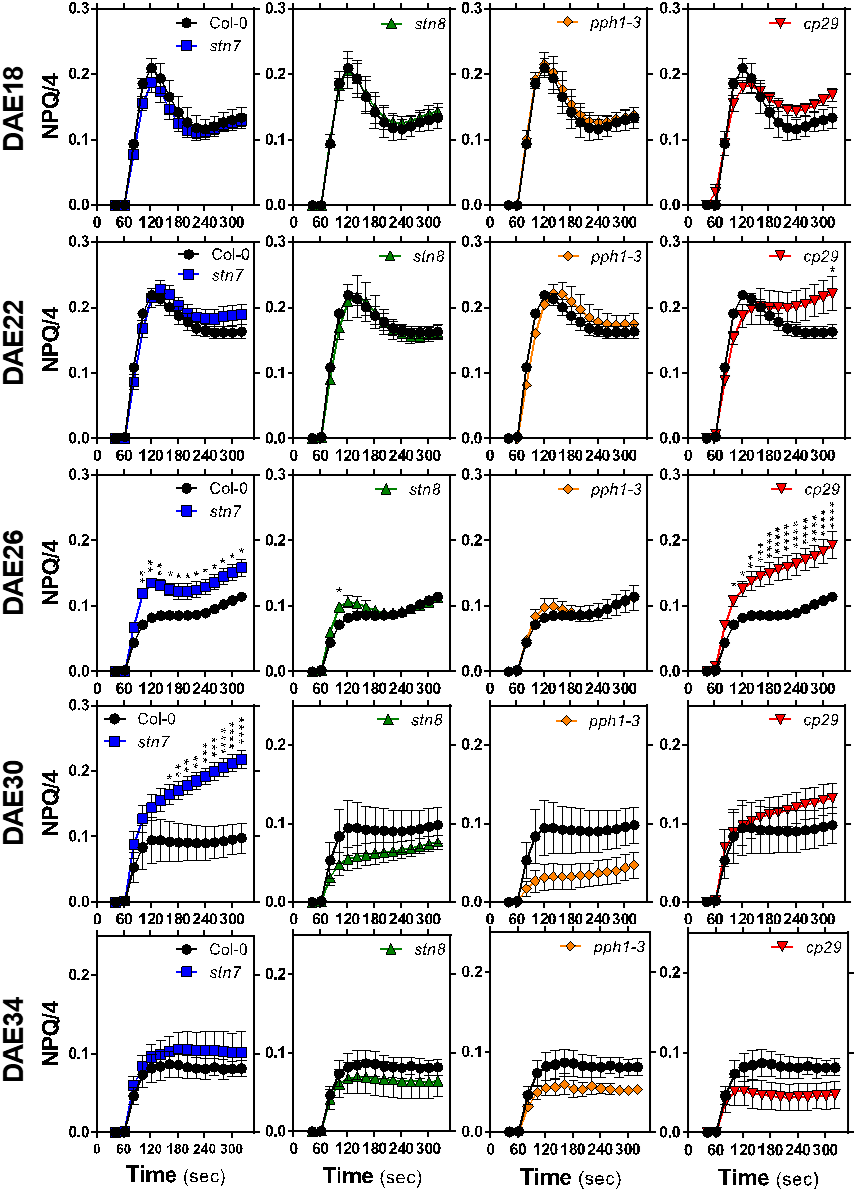


Supplementary Figure 2 Leaf age dependent nonphotochemical quenching. Data are mean ± SE from 4 to 5 biological trials. Asterisk indicates p-value after two-way ANOVA test (*, p < 0.05; **, p < 0.01; ***, p < 0.001; ****, p < 0.0001).

Supplementary Figure 3 Degradation of photosynthetic protein complexes in thylakoid membrane with leaf age. Photosynthetic protein complexes in grana stack enriched by solubilizing with DM, and stroma lamella enriched by solubilizing with digitonin were analyzed to determine their complex de-formation with leaf age. Independent biological trials were displayed. These blue native gels were full-gel images.

D1 original blot images

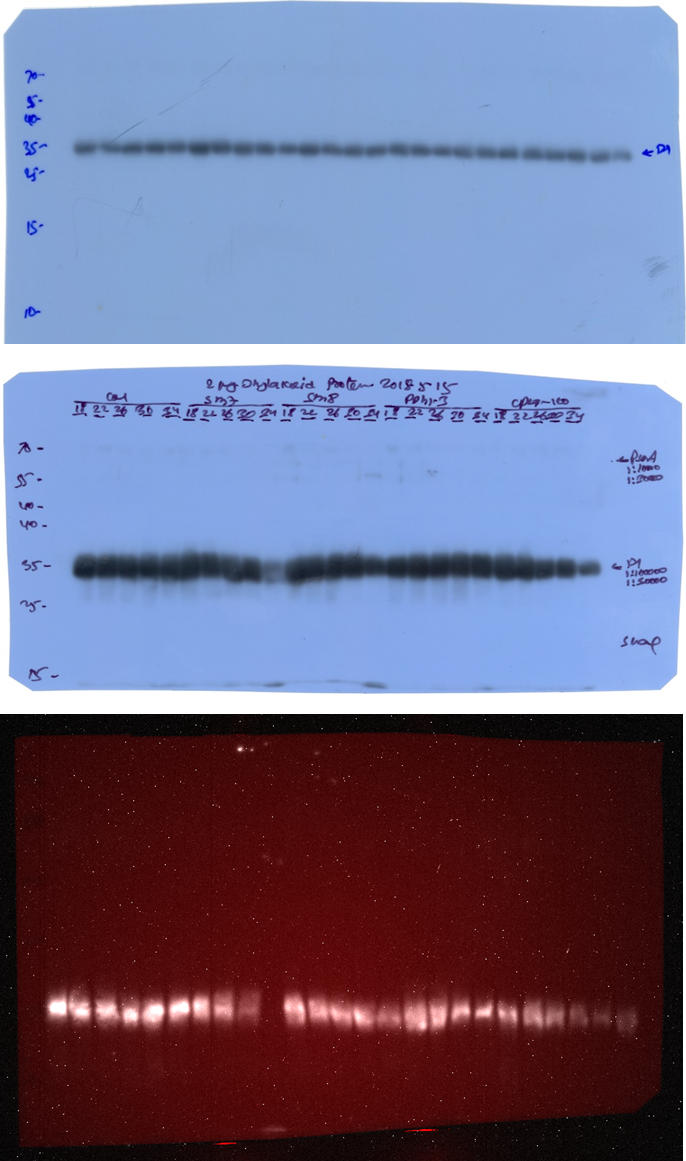


LHCB1 original blot images

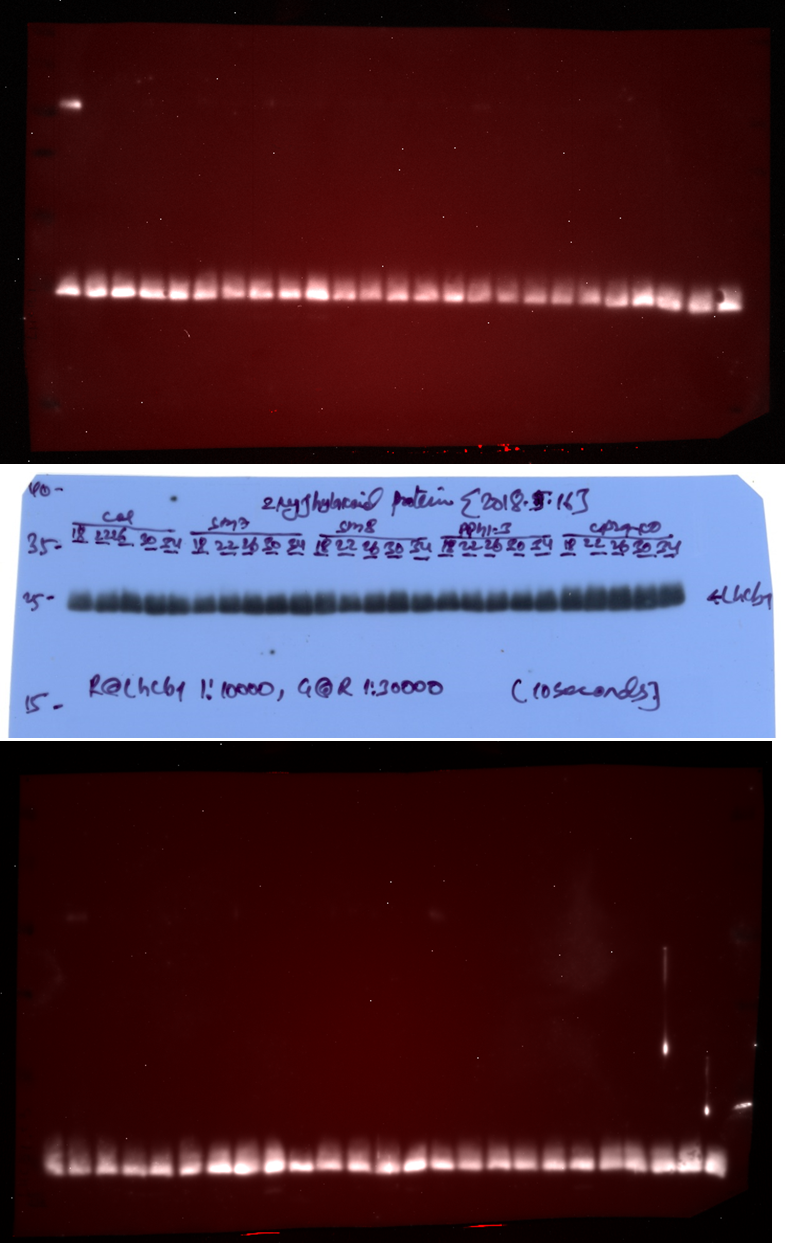


LHCB2 original blot images

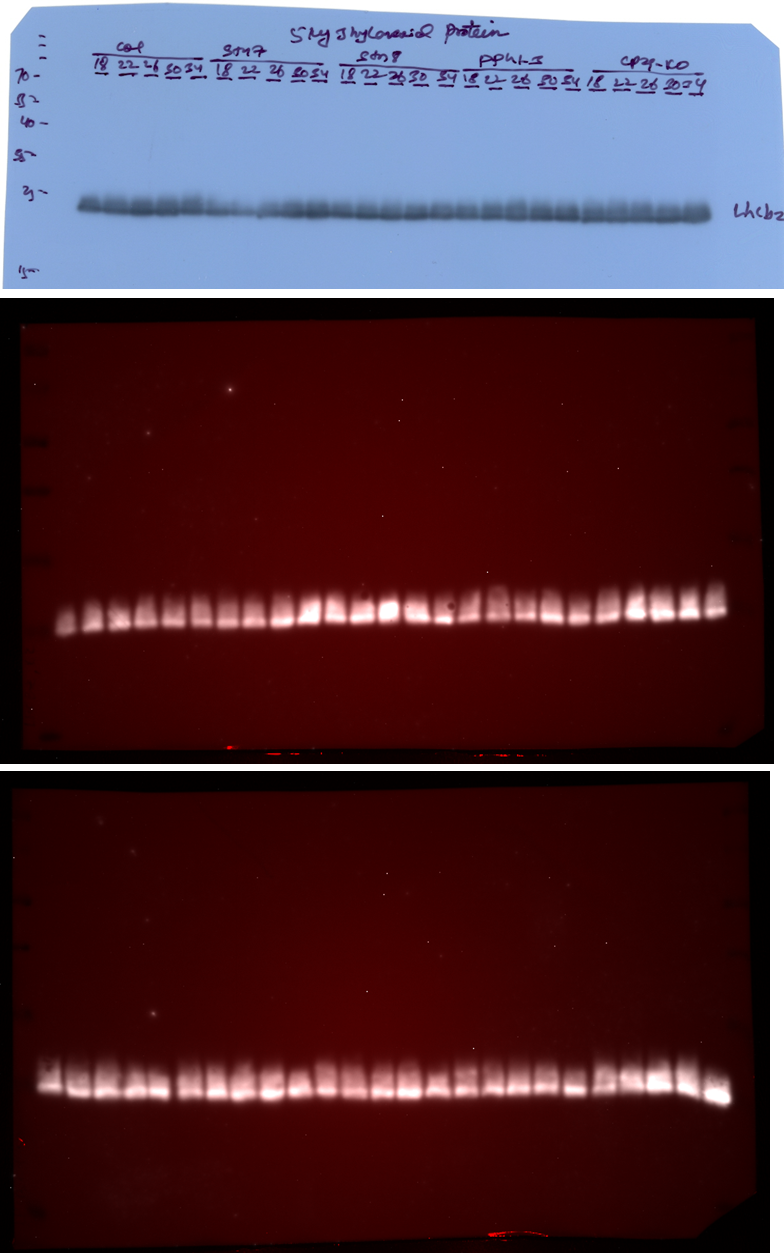


CP29 original blot images


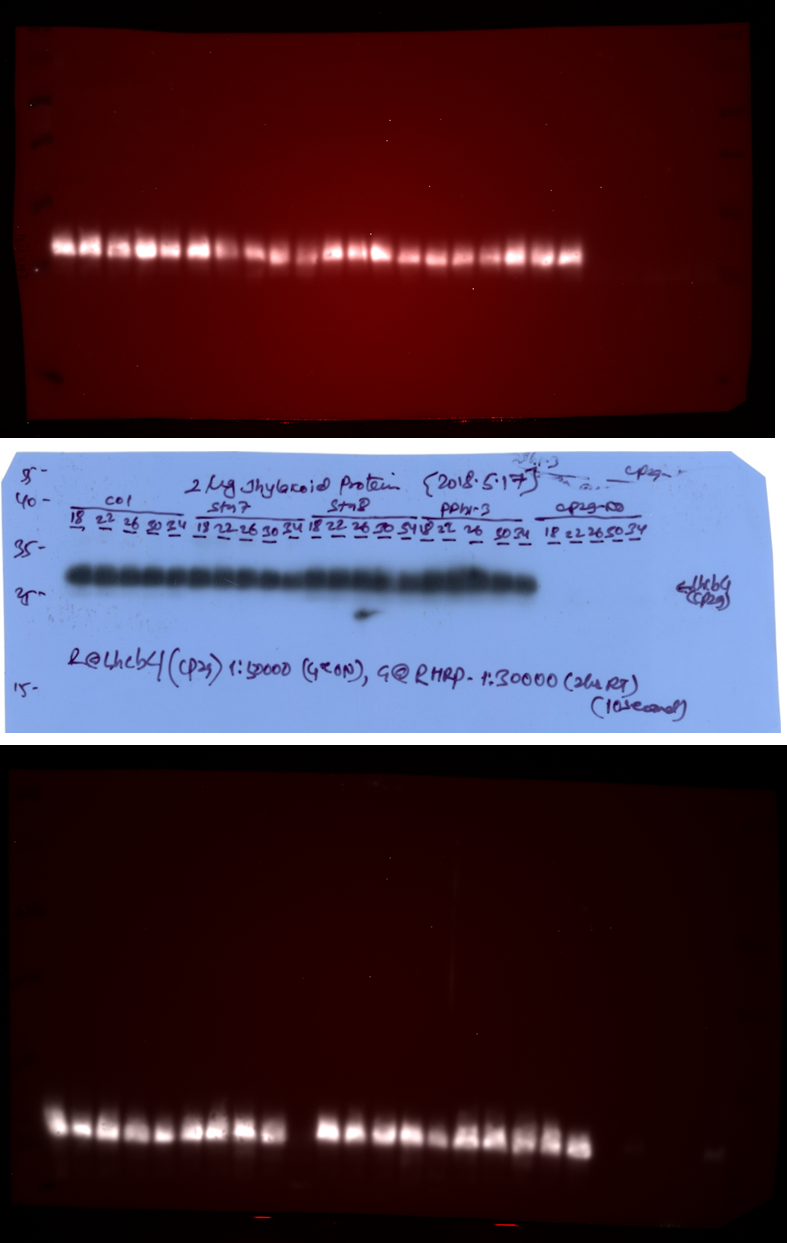


PsaA/B original blot images


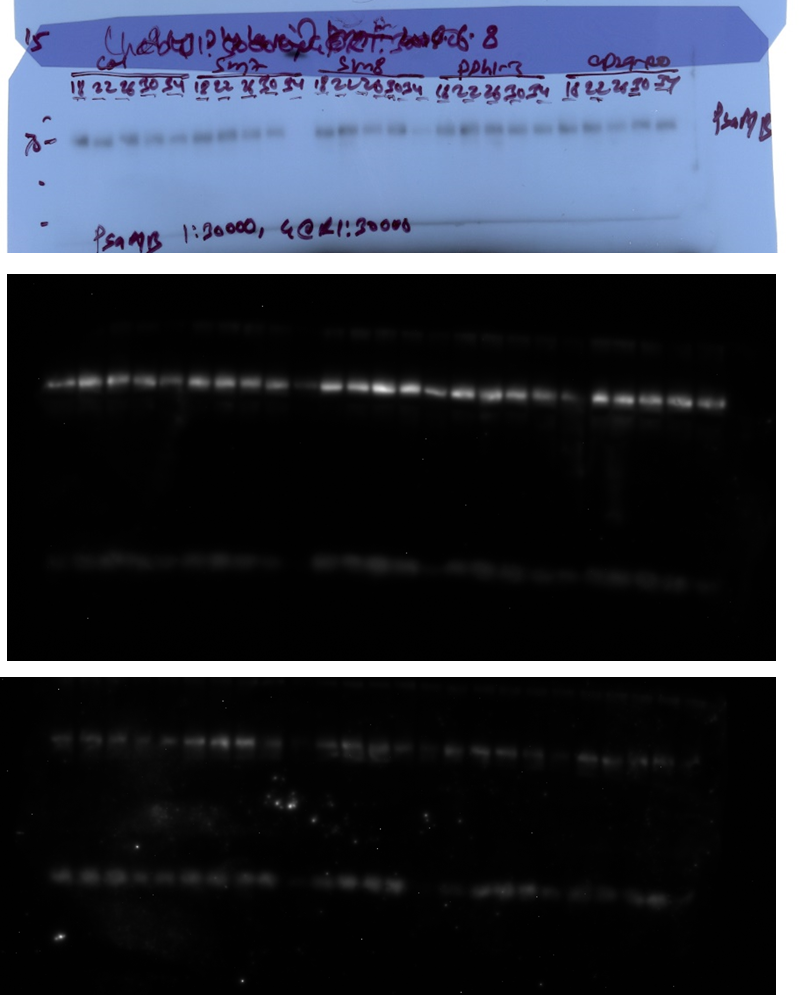


LHCA1 original blot images

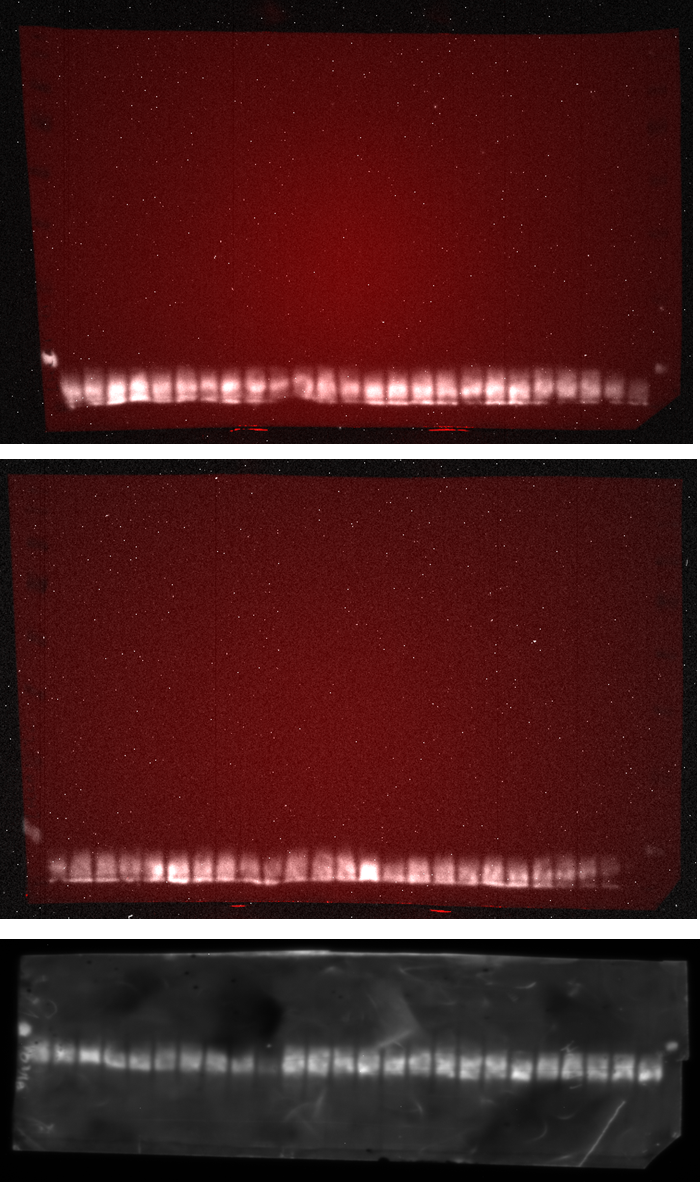


LHCA2 original blot images

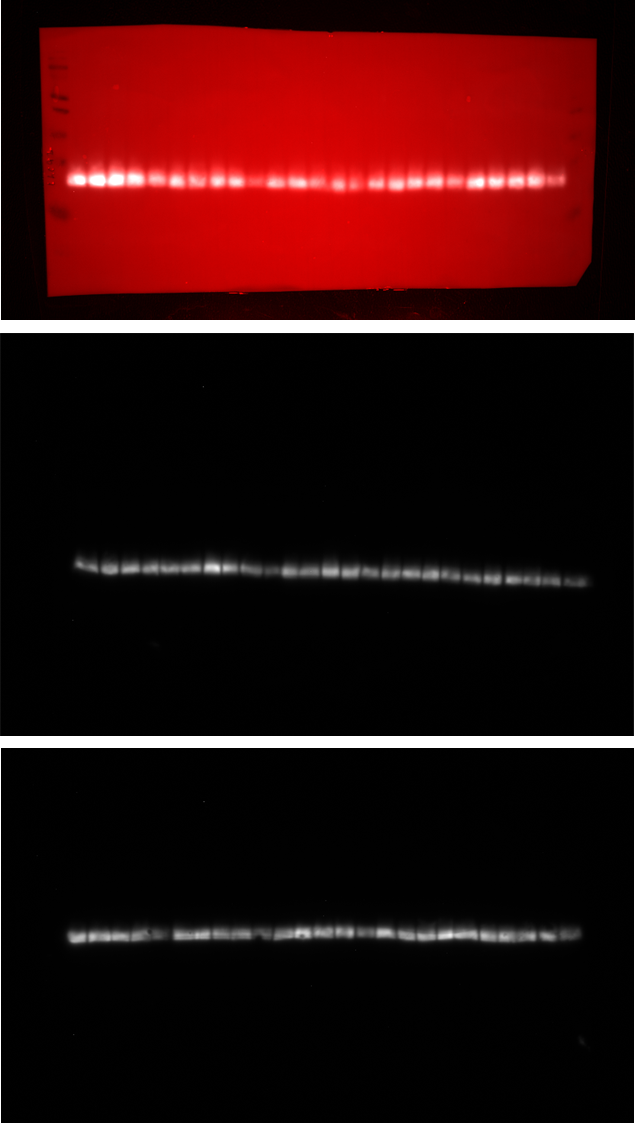


Cytb6f original blot images

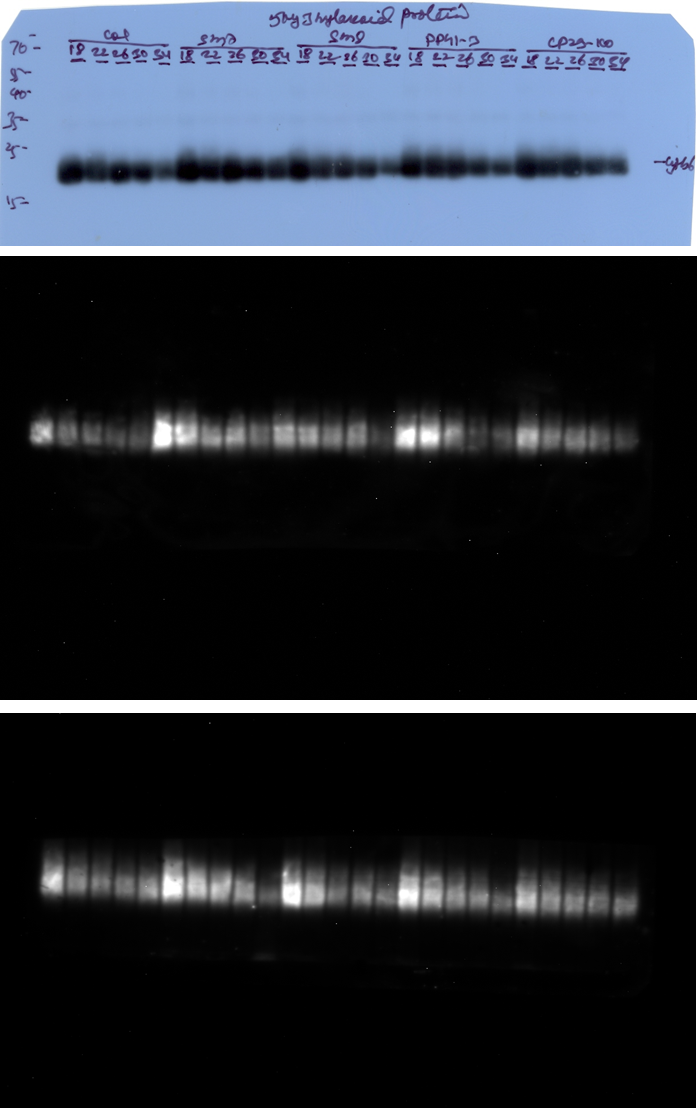


ATPase original blot images


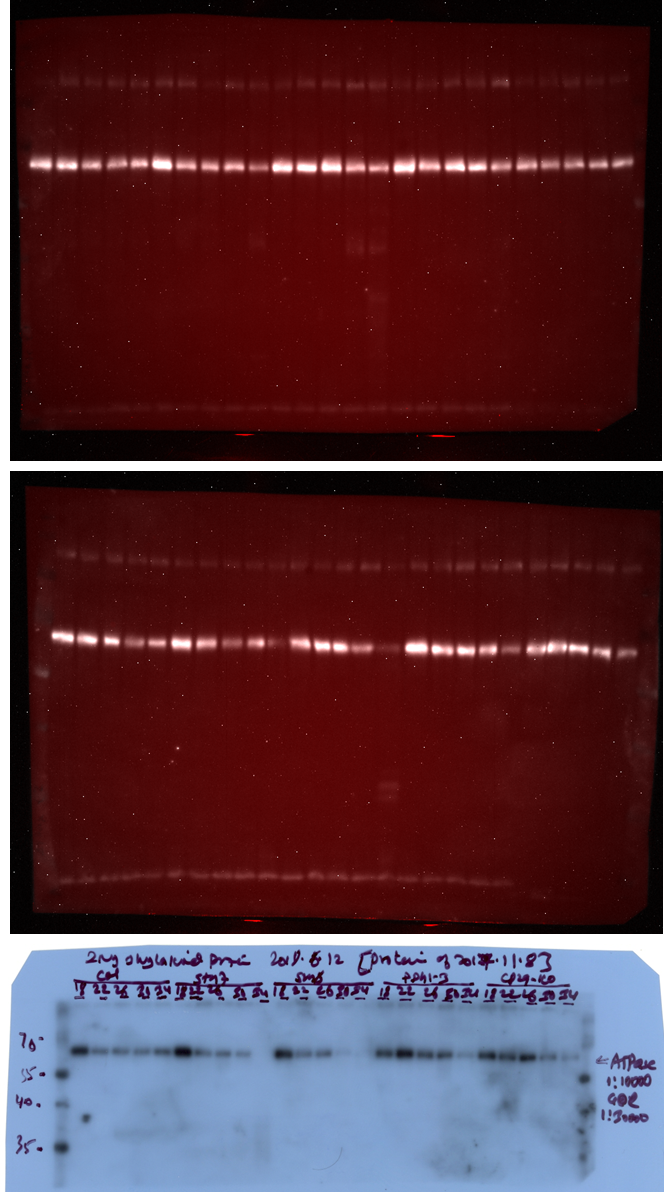


**Supplementary Figure 4 Leaf age dependent photosynthetic protein degradation.**

Original blot images for Figure 3B. Three biological trials for each protein were displays in order.


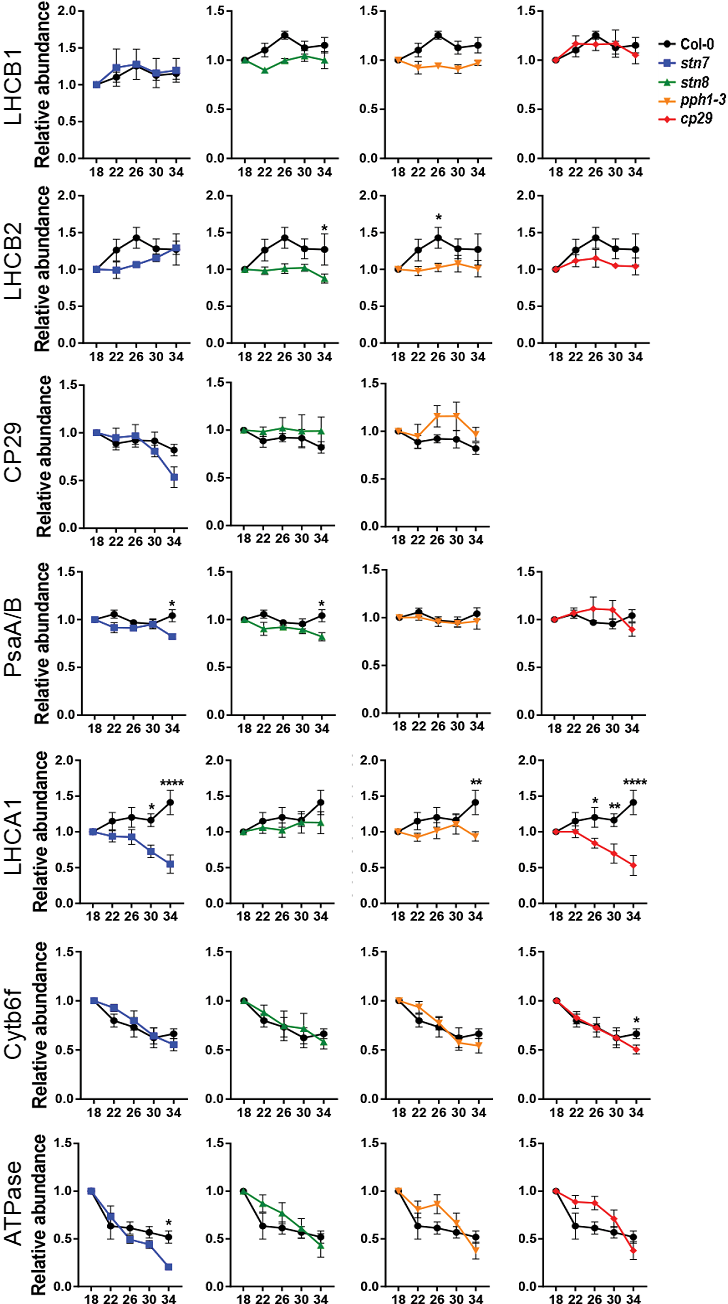


Supplementary Figure 5 Leaf age dependent photosynthetic protein degradation. Quantification of photosynthetic protein abundance from 3 biological trials. Data are mean ± SE, and p-value is displayed after the two-way ANOVA test. Asterisks indicate **, p < 0.01 and ****, p < 0.0001 comparing WT with mutants.

Supplementary Figure 6 Leaf age-dependent decrease of phosphorylation at Ser/Thr residues in key photosynthetic proteins. Independent biological trials were displayed.


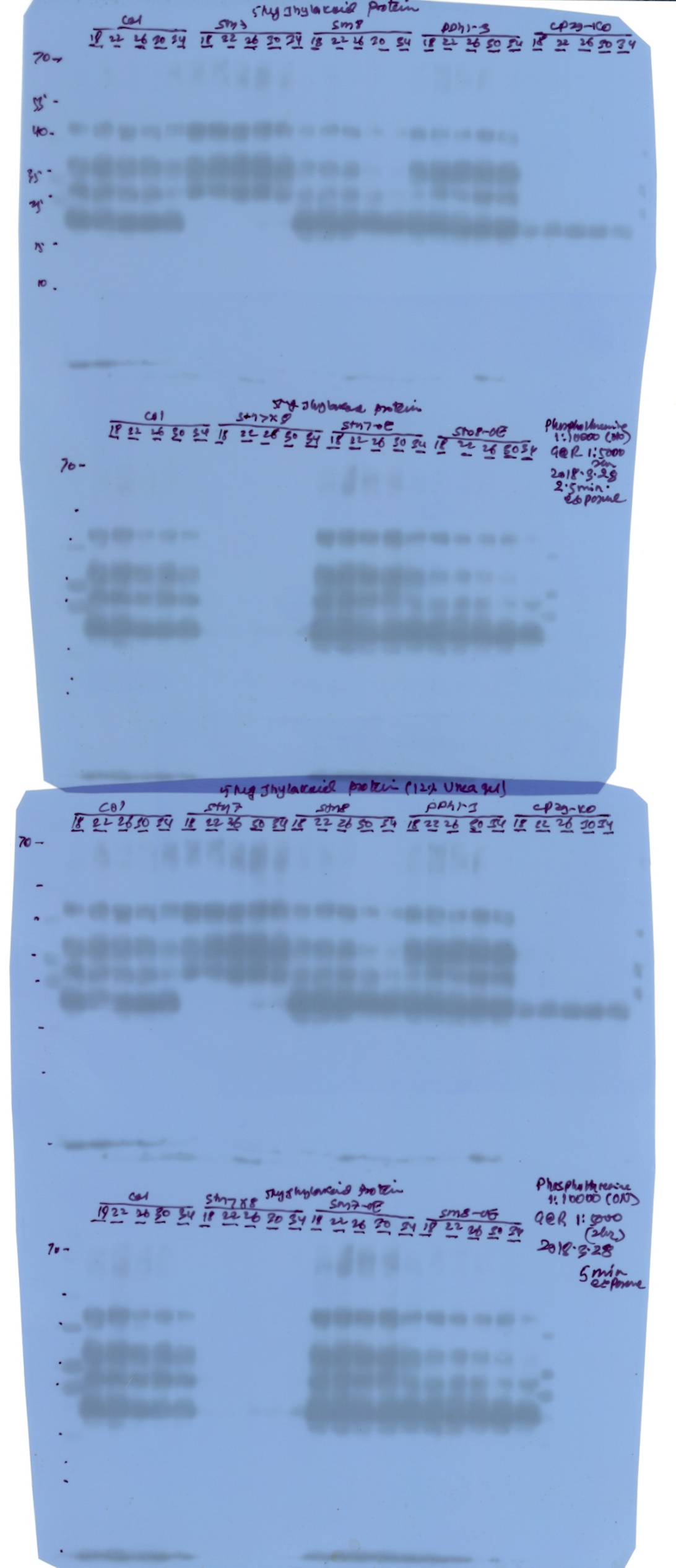


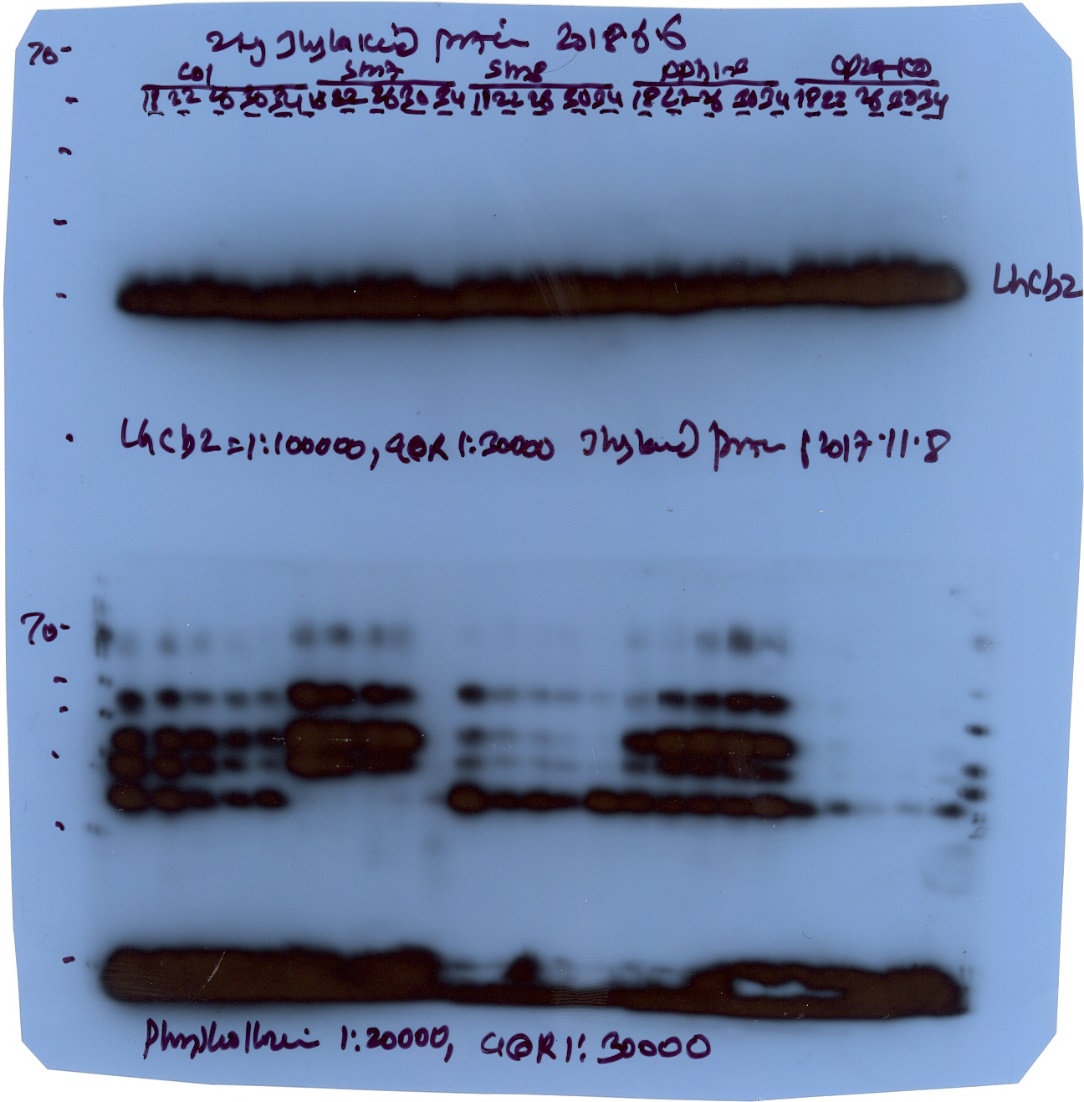


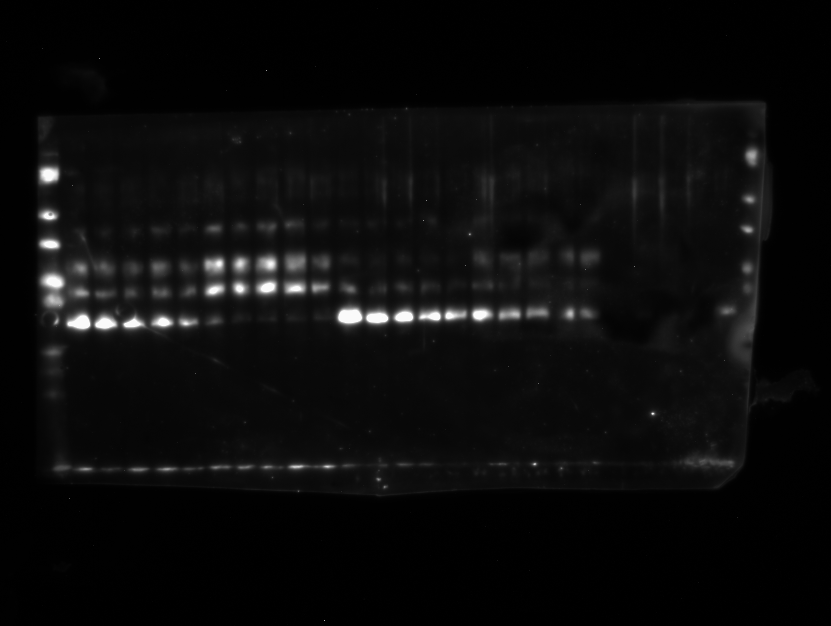


Supplementary Figure 7 Leaf age-dependent decrease of phosphorylation at Ser/Thr residues in key photosynthetic proteins. Original blot images of Figure 3D and Supplementary Figure 6. First two blots were exposed using X-ray film and the last blot were imaged by using FluorChem E system (protein simple, USA).

Supplementary Figure 8 Changes of CP29 protein phosphorylation and fragmentation in senescing leaves. Independent biological trials were displayed. Red arrows indicate phosphorylated CP29.


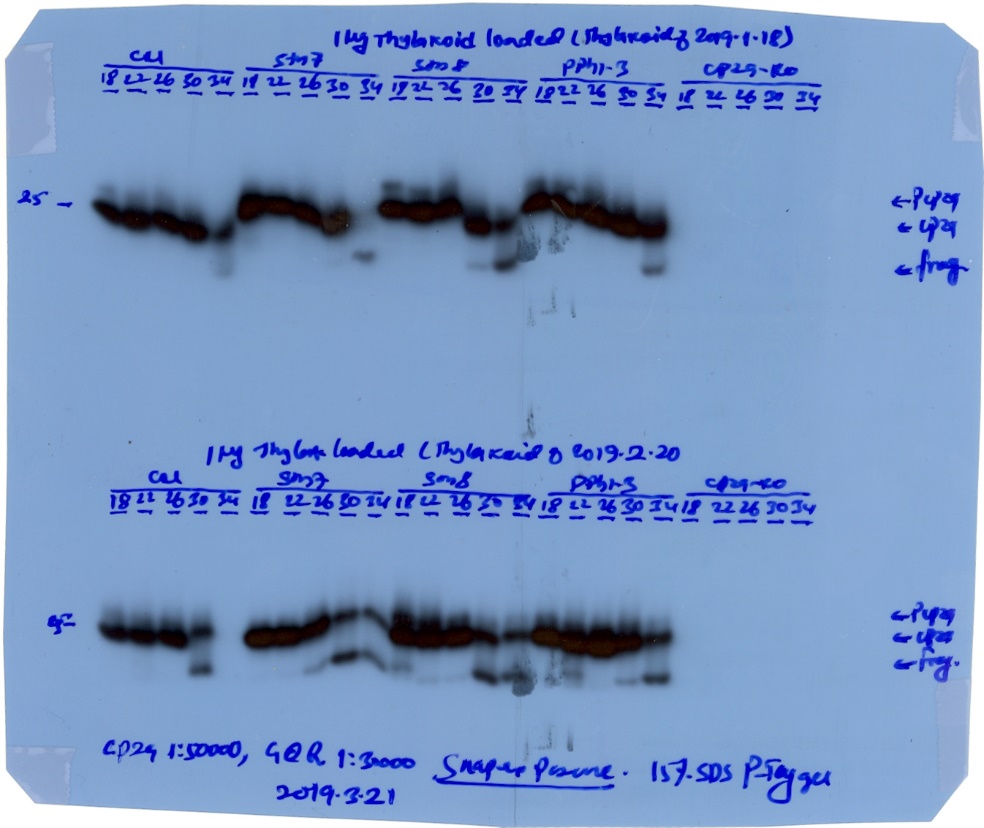


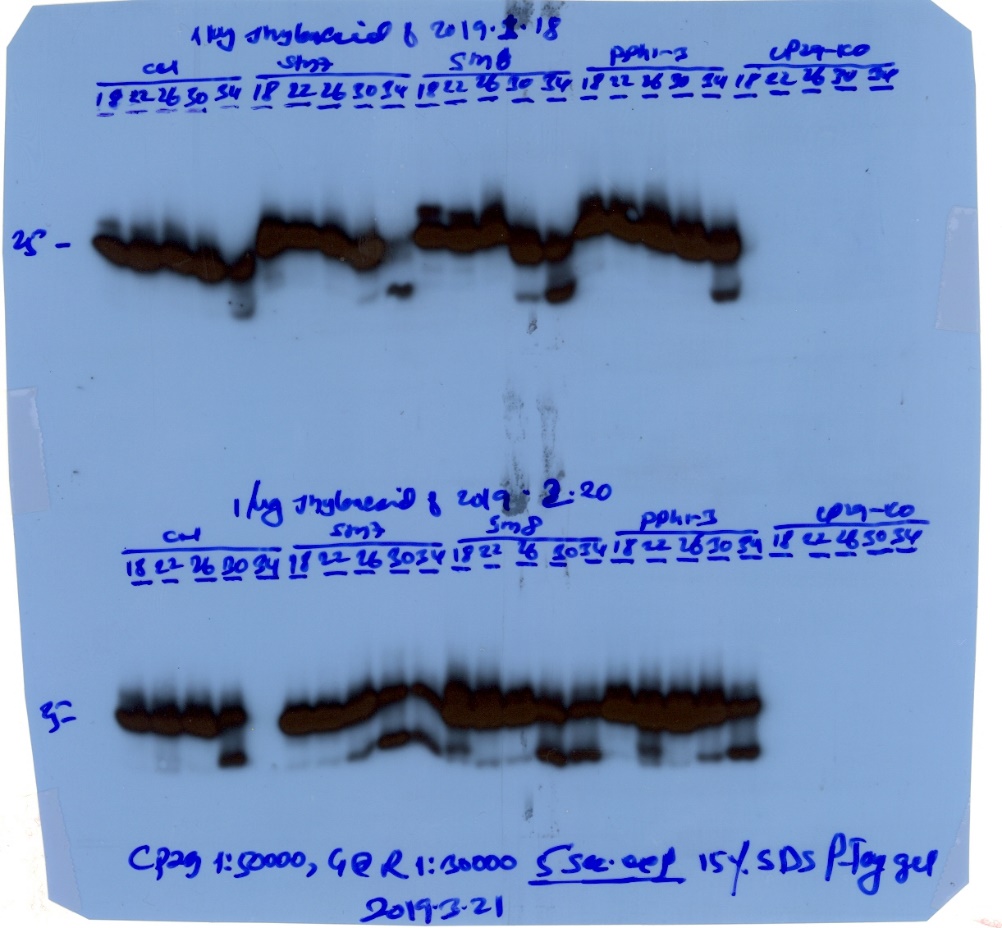


Supplementary Figure 9 Changes of CP29 protein phosphorylation and fragmentation in senescing leaves. Original blot images from two biological trials. Left and right blot are short and long exposure blot.

Supplementary Figure 10 Amino acid sequence comparison among three CP29 isoforms. CP29.1, CP29.2, and CP29.3 have 291, 288, and 277 amino acids, respectively. Red letters with yellow background represent identical sequences, blue letters with pale blue background represent conservative sequences, and black letters with green background represent block of similar sequences. Red stars on consensus sequences indicate known and conserved phosphorylation sites by STN7.

Supplementary Figure 11 Complementation of CP29 protein among CP29 transgenics. To test CP29 isoform dosage-dependent photosynthetic protein complex complementation, we screened 24 transformants per transgenic line and selected 3 independent lines based on their abundance. S, M, and W represent strong, medium, and weak expression, respectively.


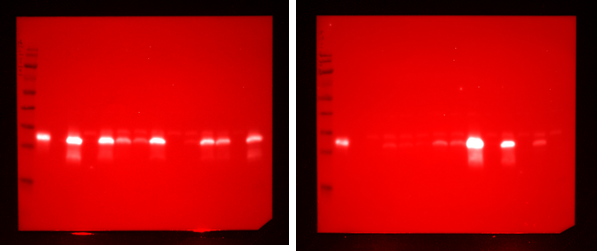
CP29.1 OE original blot image


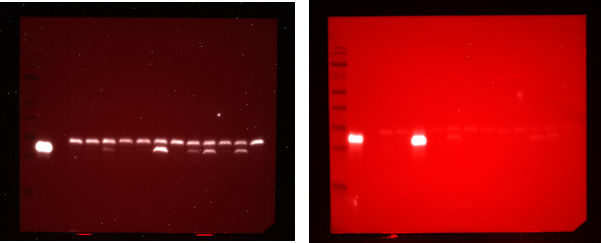
CP29.2 OE original blot image


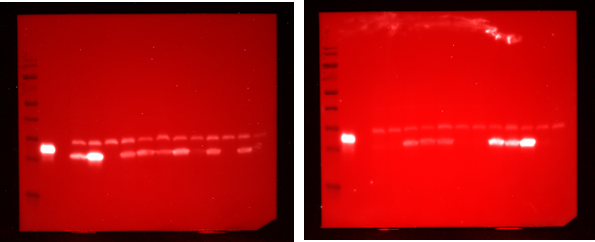

CP29.3 OE original blot image


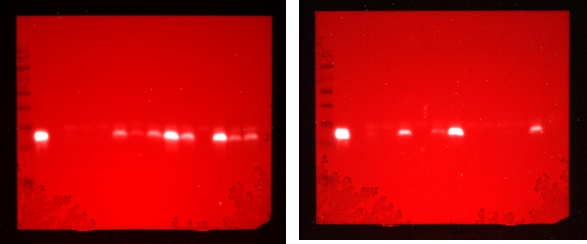

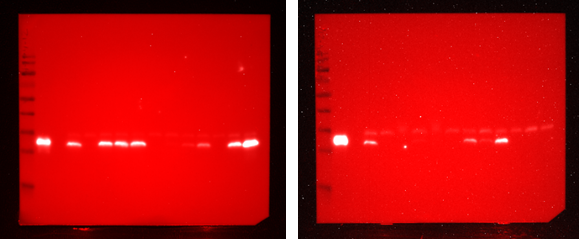
CP29.1_T112/114A_ original blot image


CP29.2 _T112/114A_ original blot image

Supplementary Figure 12 Complementation of CP29 protein among CP29 transgenics. To test CP29 isoform dosage-dependent photosynthetic protein complex complementation, we screened 24 transformants per transgenic line and selected 3 independent lines based on their abundance. S, M, and W represent strong, medium, and weak expression, respectively.
